# Dexrazoxane significantly impairs the induction of doxorubicin resistance in the human leukaemia line, K562

**DOI:** 10.1054/bjoc.2001.1697

**Published:** 2001-04

**Authors:** J M Sargent, C J Williamson, C Yardley, C G Taylor, K Hellmann

**Affiliations:** Haematology Research, Pembury Hospital, Pembury, Kent TN2 4QJ, UK

**Keywords:** dexrazoxane, doxorubicin, K562, drug resistance, MDR, P-glycoprotein

## Abstract

Dexrazoxane combined with doxorubicin (+ 5-fluorouracil + cyclophosphamide – the FAC regime) leads to a significant decrease in doxorubicin cardiotoxicity and a significant increase in median survival time for patients with advanced breast cancer responsive to FAC. The reason for this increase in survival may be due to interference with the mechanism involved in the emergence of multidrug resistance (MDR). In order to test this hypothesis, we induced resistance to doxorubicin in the K562 cell line by growing cells in increasing concentrations of doxorubicin (10–30 nM) in the presence and absence of dexrazoxane (20 nM). The doxorubicin sensitivity of all resultant sublines was measured using the MTT assay. Flow cytometry was used to assess the MDR1 phenotype, measuring P-glycoprotein expression with MRK 16 antibody and drug accumulation in the presence and absence of PSC 833 for functional P-glycoprotein. Long-term growth in doxorubicin increased the cellular resistance (IC _50_) of K562 cells in a concentration-dependent manner (r^2 ^= 0.908). Doxorubicin resistance was not induced in the presence of dexrazoxane (*P*< 0.0001) for several months. In parallel, the expression of functional P-glycoprotein was delayed after concomitant addition of dexrazoxane to the selecting medium (*P*< 0.001). Dexrazoxane did not act as a conventional modulator of P-glycoprotein. These results suggest that dexrazoxane may delay the development of MDR1, thus allowing responders to the FAC regime to continue to respond. © 2001 Cancer Research Campaign http://www.bjcancer.com


British Journal of Cancer (2001) 84(7), 959–964
© 2001 Cancer Research Campaign
doi: 10.1054/ bjoc.2001.1697, available online at http://www.idealibrary.com on IDEAL

### Dexrazoxane significantly impairs the induction of doxorubicin resistance in the human leukaemia line, K562

**JM Sargent, CJ Williamson, C Yardley, CG Taylor and K Hellmann**

Haematology Research, Pembury Hospital, Pembury, Kent TN2 4QJ, UK

**Summary** Dexrazoxane combined with doxorubicin (+ 5-fluorouracil + cyclophosphamide – the FAC regime) leads to a significant decrease in doxorubicin cardiotoxicity and a significant increase in median survival time for patients with advanced breast cancer responsive to FAC. The reason for this increase in survival may be due to interference with the mechanism involved in the emergence of multidrug resistance (MDR). In order to test this hypothesis, we induced resistance to doxorubicin in the K562 cell line by growing cells in increasing concentrations of doxorubicin (10–30 nM) in the presence and absence of dexrazoxane (20 nM). The doxorubicin sensitivity of all resultant sublines was measured using the MTT assay. Flow cytometry was used to assess the MDR1 phenotype, measuring P-glycoprotein expression with MRK 16 antibody and drug accumulation in the presence and absence of PSC 833 for functional P-glycoprotein. Long-term growth in doxorubicin increased the cellular resistance ($IC_{50}$) of K562 cells in a concentration-dependent manner ($r^2 = 0.908$). Doxorubicin resistance was not induced in the presence of dexrazoxane ($P < 0.0001$) for several months. In parallel, the expression of functional P-glycoprotein was delayed after concomitant addition of dexrazoxane to the selecting medium ($P < 0.001$). Dexrazoxane did not act as a conventional modulator of P-glycoprotein. These results suggest that dexrazoxane may delay the development of MDR1, thus allowing responders to the FAC regime to continue to respond. © 2001 Cancer Research Campaign http://www.bjcancer.com

**Keywords**: dexrazoxane; doxorubicin; K562; drug resistance; MDR; P-glycoprotein

Despite major advances in drug development, support therapy and adjuvant treatment, overall survival in advanced breast cancer continues to be discouragingly low. Drug resistance remains one of the major problems in this disease. Development of the multidrug resistance (MDR) phenotype is particularly challenging because tumours become cross-resistant to multiple, chemically unrelated cytotoxic agents. Expression of the *MDR1* gene, characterized by the presence of the 170 kD transmembrane transporter P-glycoprotein (Pgp), is often low prior to treatment but is frequently increased during the progression of disease and most notably after chemotherapy (Chaudhary and Roninson, 1993). Indeed, increased Pgp expression has been repeatedly observed in samples from patients with breast cancer (Trock et al, 1997). Conventional approaches to overcoming this resistance have involved the concomitant administration of resistance modulators, usually as second line therapy. These agents include the more potent second generation analogues such as SDZ PSC 833 (PSC 833) which is capable of circumventing MDR (Advani et al, 1999). Clinical trials utilizing these agents have, however, been disappointing particularly in solid tumours (Kaye, 1999) and a novel approach to the problem may be required. Instead of treating resistance after it has occurred, perhaps it may be more pertinent to attempt to prevent its emergence right from the onset of treatment. There is clinical evidence to suggest that the chelating agent, dexrazoxane (DXRz) may have this effect.

*Received 12 September 2000*
*Revised 20 December 2000*
*Accepted 2 January 2001*

*Correspondence to:* JM Sargent

Dexrazoxane protects against the cardiotoxicity of doxorubicin and when combined with the FAC regime (5-fluorouricil, doxorubicin and cyclophosphamide) not only leads to a significant decrease in cardiotoxicity but also to a substantial increase in overall median survival time for patients with advanced breast cancer responsive to FAC (Swain et al, 1997). This survival advantage could be explained by a reduction in the emergence of MDR since dexrazoxane has been reported to inhibit topoisomerase II and to potentiate the efficacy of a number of antitumour agents (Hasinoff et al, 1998) in addition to its cardioprotective effect. At present, any common factor connecting these events remains speculative.

The aim of this in-vitro study was to attempt to mimic the clinical observation that the combination of dexrazoxane with doxorubicin leads to increased efficacy, with particular interest in the development of drug resistance. Doxorubicin resistance was induced in the presence and absence of clinically achievable levels of dexrazoxane in the human cell line, K562. The K562 line was chosen, as it is a robust model which develops an MDR1 phenotype after a relatively short period of exposure to doxorubicin (Kato et al, 1990). The expression and function of P-glycoprotein was measured along with cellular sensitivity in order to assess emerging MDR1 in the resultant sublines.

### MATERIALS AND METHODS

### Cell lines

K562 is a human leukaemia cell line that was derived from a patient with chronic myeloid leukaemia in blast cell crisis. By stepwise increasing the concentration of doxorubicin, this cell line develops an MDR1 phenotype within a few weeks (Kato et al, 1990). K562 cells have a doubling time of approximately 16 hours, were maintained in RPMI 1640 culture medium plus 10% FCS, 100 1U $ml^{-1}$ penicillin, 100 μg $ml^{-1}$ streptomycin (all from Sigma Aldridge, UK) and were passaged every 48 hours to $1.0 \times 10^5$ cells $ml^{-1}$.

A pilot study was carried out in order to optimize the concentrations for long-term exposure to dexrazoxane (Cardioxane, Chiron, UK) and doxorubicin. We tested dexrazoxane at a range of concentrations up to 5 μM and established that concentrations up to 200 nM were commensurate with long-term growth. Doxorubicin resistant sublines were then generated by incubating K562 parental cells at $1.0 \times 10^5$ cells $ml^{-1}$ in the presence of sublethal concentrations of doxorubicin ± dexrazoxane. The starting concentration used for doxorubicin was 10 nM and it was possible to increase this stepwise by 10 nM amounts to 40 nM in the presence of 20 nM dexrazoxane. Cells selected in doxorubicin without dexrazoxane showed typical signs of drug damage manifested by cell swelling, alterations in cell shape and granularity. This became detectable after 4 days and when the cells had adapted to this environment (approximately 2–4 weeks) the concentration of doxorubicin was increased. The cells were examined microscopically every 48 hours before passaging and if low growth and/or increasing background debris were seen, cells were maintained in the same drug concentration for a further week. Preliminary results of this pilot study were extremely encouraging and suggested a significant impairment of the MDR1 phenotype in cells selected in doxorubicin + dexrazoxane.

A further experiment was carried out using the optimum concentrations of dexrazoxane and doxorubicin established in the pilot study. In this experiment, cells were selected in increasing concentrations of doxorubicin up to 30 nM ± 20 nM dexrazoxane. Cells selected in 15 and 20 nM ($K562DOX_{15}$, $K562DOX_{20}$) were frozen in liquid nitrogen in RPMI 1640 plus 20% FCS and 10% DMSO to enable batch testing for cytotoxicity and MDR markers alongside cells selected in 30 nM doxorubicin ($K562DOX_{30}$). K562 parental cells, the MDR-positive $K562DOX_{100}$ subline maintained in 100 nM doxorubicin and cells grown in dexrazoxane alone at the above concentrations were included as controls.

### Cytotoxicity studies

The doxorubicin cytotoxicity of each of the resultant sublines grown in the presence and absence of dexrazoxane was measured on 2 separate occasions. Cells from all sublines were continually exposed to 8 concentrations (0.2–10 μM) of doxorubicin for 48 h at 37˚C, 5% $CO_2$. Cells incubated in culture medium without drug, served as controls. The MTT assay was used to assess cell survival after drug exposure (Sargent and Taylor, 1989) and the $IC_{50}$ (concentration of drug causing 50% growth inhibition) was calculated for each experiment.

Doxorubicin cytotoxicity was also measured in the presence (at a fixed concentration of 2 μM) of the MDR modulator PSC 833 (gift from Novartis, UK). A sensitization ratio of the $IC_{50}$ for doxorubicin over $IC_{50}$ for doxorubicin + PSC 833 gave a measure of modulation effect.

In order to test whether dexrazoxane acts as an MDR modulator in the short term, K562 cells and $K562DOX_{15,30 \text{ and } 100}$ sublines were exposed to doxorubicin as above ± dexrazoxane at 20 nM or 4 μM (non-toxic concentration) for 48 hours. Cells from each sub-line exposed to dexrazoxane at the appropriate concentration were used as controls. Sensitization ratios were calculated as above.

### MDR1 gene expression

RT-PCR was used to determine *MDR1* gene expression in all sublines. The single step method used for isolation of RNA was based on that of Chomczynski and Sacchi (1987) using Triazol reagent (Life Technologies, UK). Total RNA was reverse transcribed using Superscript II (Life Technologies, UK) and random hexanucleotides (Pharmacia), according to manufacturers' instructions. PCR was performed on cDNA as described by Yanagisawa et al (1999). The sequences of the *MDR*1 primers (Oswel DNA Services, Southampton, UK) were as previously reported (Bordow et al, 1994). A 3 min initial denaturation step at 94˚C was followed by 30 cycles of 45 s at 94˚C, 45 s at 55˚C and 90 s at 72˚C ending with 10 min at 72˚C (Touchdown Thermocycler, Hybaid, UK). An RNA free negative control was run and $\beta_2$-microglobulin was used as an internal control for DNA loading. PCR products were visualized after gel electrophoresis (1% agarose) by UV translumination using ethidium bromide staining. Results were recorded using a Gelcam Polaroid camera (Helena Biosciences, UK).

### P-glycoprotein expression

The P-glycoprotein expression of all cell lines was measured by flow cytometry with the MRK 16 antibody (TCS Biologicals, UK). Indirect immunofluorescence was carried out utilizing a FITC-labelled goat anti-mouse secondary antibody (Beckman Coulter, UK). An isotype matched control (IgG2a, Sigma Aldrich, UK) was run with every test at the same IgG concentration as the test antibody to measure any non-specific binding. Cells ($0.5–2 \times 10^6$) were incubated in MRK 16 (5 μg $ml^{-1}$) for 1 h at room temperature. After washing × 2, the FITC-labelled secondary antibody was added to all tubes and allowed to react for 45 min at room temperature in the dark. After washing × 2, cells were kept on ice until analysed using a Coulter Epics XL flow cytometer equipped with an argon laser with an excitation wavelength of 488 nm. Results were expressed as the ratio of the mean fluorescence associated with the test over that for the control. Therefore, results >1.0 indicate the presence of P-glycoprotein.

### Drug accumulation

Using flow cytometry, the accumulation of daunorubicin (Cerubidin, Rhone-Poulenc Rorer, UK) was measured in the presence and absence of the MDR-modulating agent PSC 833 to indicate the presence of functional P-glycoprotein. Daunorubicin, an analogue of doxorubicin, was used because it is a good substrate for P-glycoprotein and we have previous experience of undertaking functional analysis of P-glycoprotein using this methodology (Elgie et al, 1999). Cells ($5 \times 10^5$) from all lines were incubated with and without PSC 833 (2 μM) for 1 hour at 37˚C, 5% $CO_2$. Daunorubicin (5 μM) was added throughout for a further hour incubation. Cells were then washed × 2 in ice cold PBS and kept on ice until required for flow cytometric analysis. Drug accumulation was measured as mean fluorescence units (MFUs).

### Statistics

The effect of dexrazoxane on the generation of resistance to doxorubicin, the ability of dexrazoxane or PSC 833 to modulate

 *© 2001 Cancer Research Campaign*

P-gp were all analysed using Student's *t* test. The relationship between doxorubicin in the growth medium and cellular resistance ($IC_{50}$) was analysed using the Pearson *r* correlation coefficient.

### RESULTS

### Doxorubicin cytotoxicity

There was a linear relationship between increasing concentration of doxorubicin in the selecting medium (15–30 nM) and increasing doxorubicin $IC_{50}$ ($r^2 = 0.908$). The addition of 20 nM dexrazoxane to the selecting medium impaired this increase in $IC_{50}$ (Table 1, $P < 0.0001$).

When the modulating agent PSC 833 was added to the cytotoxicity assay, the sublines cultured in >15 nM doxorubicin showed high sensitization ratios indicating the presence of MDR (Table 1). Ratios obtained from cells incubated in doxorubicin + dexrazoxane were all ≤1.0 with no indication of MDR. Sensitization ratios obtained from cells grown in dexrazoxane alone were not significantly different from parental cells ($P = 0.686$).

### Dexrazoxane as a modulator of P-gp

Table 2 shows that there was no significant increase in doxorubicin sensitivity after 48 hour co-incubation of $K562DOX_{30}$ or $K562DOX_{100}$ cells with dexrazoxane either at 20 nM or 4 μM, a concentration found non-toxic after short-term exposure. These results suggest that dexrazoxane does not overcome MDR once present.

### Growth characteristics and appearance of sublines

Poor growth was seen for cells incubated in doxorubicin + dexrazoxane. On microscopic examination, many apoptotic changes could be noted with nuclear fragmentation, blebbing and a marked variation in cell size from cell fragments to large swollen cells. These cells remained sensitive to doxorubicin but were being cultured in sublethal concentrations of doxorubicin, approximately a tenth of $IC_{50}$. The appearance of cells selected in doxorubicin alone, however, recovered within 2 weeks, when they closely resembled the parental cells. Cells grown in dexrazoxane alone always seemed healthy and appeared no different from the parental cells having the same doxorubicin sensitivity as the parental line and lacking the MDR1 phenotype.

### MDR1 gene expression

Figure 1 shows that MDR1 mRNA was increased when the doxorubicin concentration in the selecting medium was >15 nM. In the presence of dexrazoxane, this increase in gene expression was abolished in cells selected in 20 nM doxorubicin ($K562DOX_{20}$) and markedly reduced in cells selected in 30 nM ($K562DOX_{30}$) doxorubicin. The $\beta_2$-microglobulin results confirmed that RNA levels were comparable throughout all lines.

### P-glycoprotein expression

The P-glycoprotein expression of the sublines is shown in Figure 2. Without dexrazoxane, P-glycoprotein appears to quickly reach maximal expression after selection in >15 nM doxorubicin. The addition of dexrazoxane to the selecting medium prevented the expression of P-glycoprotein ($P < 0.001$) for several months.

### Drug accumulation ± PSC 833

Drug accumulation was assessed after incubating cells from all lines in daunorubicin. Culture of cells in >15 nM doxorubicin significantly reduced intracellular daunorubicin content. Concomitant addition of dexrazoxane to the selecting medium

**Table 1** Effect of selection in doxorubicin ± dexrazoxane on doxorubicin $IC_{50}$, and modulation by PSC 833

| DOX in selecting medium (nM) | Dox $IC_{50}$ (μM) | | Sensitization ratio (DOX $IC_{50}$/DOX+PSC $IC_{50}$) | |
|---|---|---|---|---|
| | –DXRz | +DXRz | –DXRz | +DXRz |
| 0 | 0.22 ± 0.06 | 0.34 ± 0.06 | 0.51 ± 0.16 | 0.63 ± 0.2 |
| 15 | 0.38 ± 0.14 | 0.5 ± 0.1 | 0.76 ± 0.31 | 0.98 ± 0.15 |
| 20 | 5.31 ± 1.06 | 0.34 ± 0.0<br>*P* = 0.04 | 9.35 ± 1.87 | 0.68 ± 0.09<br>*P* = 0.044 |
| 30 | 8.57 ± 0.03 | 0.58 ± 0.25<br>*P* < 0.0001 | 12.17 ± 0.34 | 0.93 ± 0.19<br>*P* = 0.001 |
| 100 | 7.83 ± 0.43 | nt[a] | 14.91 ± 0.08 | nt |

[a]nt = not tested. Results expressed as mean of 2 separate experiments ± SE.

**Table 2** Dexrazoxane does not modulate P-glycoprotein in the short term

| DOX in selecting medium (nM) | DOX $IC_{50}$ (μM) | DOX + DXRz (20 nM) $IC_{50}$ (μM) | DOX + DXRz (4 μM) $IC_{50}$ (μM) |
|---|---|---|---|
| 0 | 0.42 ± 0.04 | 0.37 ± 0.08 | 0.41 ± 0.17 |
| 15 | 0.59 ± 0.1 | 0.49 ± 0.03 | 0.58 ± 0.08 |
| 30 | 7.07 ± 1.05 | 6.72 ± 2.42 | 6.37 ± 1.21 |
| 100 | 8.52 ± 2.67 | 8.15 ± 2.18 | 9.29 ± 2.41 |

Results expressed as mean of 2 separate experiments ± SE.

© 2001 Cancer Research Campaign

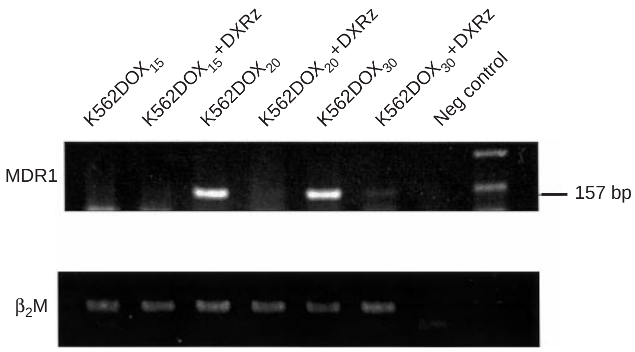

**Figure 1** *MDR1* gene expression in K562 sublines after selection in doxorubicin (DOX) ± dexrazoxane (DXRz, top panel), $\beta_2$ microglobulin controls (bottom panel)

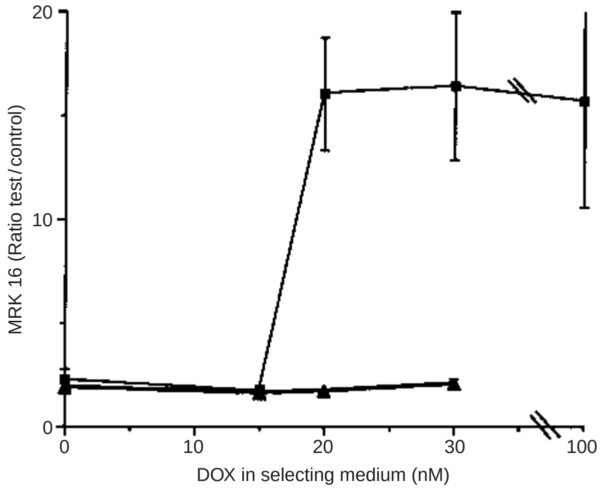

**Figure 2** P-glycoprotein expression of cells selected in increasing concentrations of doxorubicin in the presence (triangles and thick line) and absence (squares and thin line) of 20 nM dexrazoxane, $P < 0.001$. Results were expressed as the ratio of the mean fluorescence associated with cells stained with MRK 16 over that for cells stained with an isotype matched control. Data points represent the mean of 2 separate experiments ± SE

abolished this reduction (Figure 3A) ($P < 0.001$). The intracellular daunorubicin content was also measured in the presence of the MDR modulator PSC 833 to give a measure of functional P-glycoprotein. Results shown in Figure 3B suggest that the reduction in daunorubicin content found in cells selected in doxorubicin >15 nM was MDR related as PSC 833 completely inhibited this effect.

### Time course of induction of MDR1 phenotype

K562DOX$_{30}$ cells were cultured in 30 nM doxorubicin ±dexrazoxane for a period of several months (Figure 4). After approximately 5 months, weak expression of the *MDR*1 gene was seen for cells selected in both agents (Fig. 1). This was soon followed by an increase in P-gp expression which reached a peak around 8 months. The increased P-gp expression was accompanied by an attendant fall in intracellular drug accumulation and eventually by a steep rise in doxorubicin IC$_{50}$.

### DISCUSSION

The clinical observation of increased survival in breast cancer patients treated with the FAC regime plus the cardioprotectant dexrazoxane is particularly interesting because this difference in survival remained even when patients were censored on a cardiac event (Swain et al, 1997). This suggests that this beneficial effect was caused by something other than cardioprotection. It is unlikely to be higher dose chemotherapy due to reduced toxicity since the survival benefit of dose-intensive therapy has yet to be shown in breast cancer (Weiss, 1999). It is possible, therefore, that dexrazoxane is impairing the emergence of drug resistance and the results of our study support this theory. We found that dexrazoxane significantly delayed the induction of MDR1 in the K562 human cell line for several months. The effect was repeatable as our initial pilot study showed similar results. In addition, the ratio of dexrazoxane to doxorubicin commonly given for cardioprotection is 10:1. We have found, however, that dexrazoxane significantly prevents MDR at a ratio of 1:1.

If dexrazoxane already with proven effect as a cardioprotectant, also has the added effect of preventing or delaying the emergence of drug resistance as our results suggest, it then becomes an indispensable part of the cytotoxic armoury. Very few studies have addressed the approach of resistance prevention, most being concerned with resistance reversal. Sikic et al (1997) have shown that the MDR modulator PSC 833 can decrease the mutation rate for resistance to doxorubicin by suppressing the activation of the *MDR1* gene and the appearance of MDR mutants. Our findings of reduced MDR1 expression in sublines grown in the presence

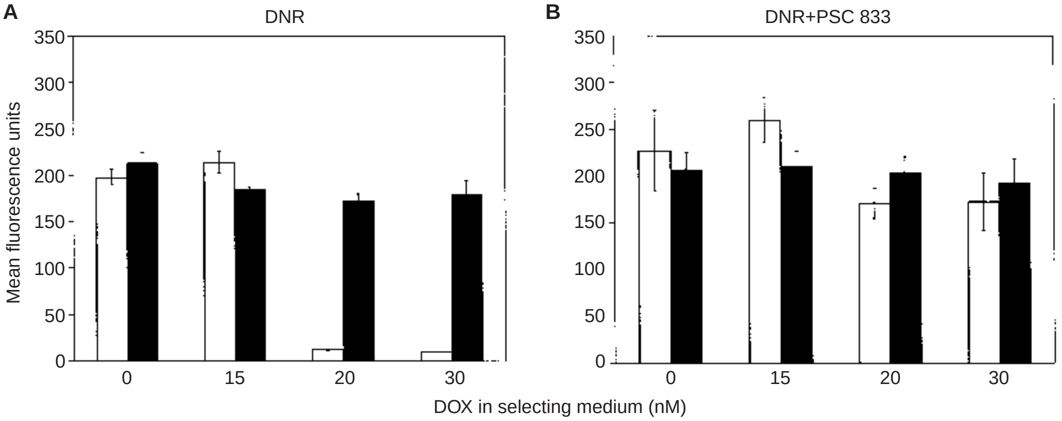

**Figure 3** (**A**) Intracellular daunorubicin concentration of cells selected in increasing concentrations of doxorubicin in the presence (black columns) and absence (white columns) of 20 nM dexrazoxane, $P < 0.001$. (**B**) Effect of concomitant incubation in PSC 833 on intracellular daunorubicin concentration. Results were expressed as mean fluorescence units after incubation in 5 µM daunorubicin. Columns represent the mean of 2 separate experiments ± SE

*© 2001 Cancer Research Campaign*

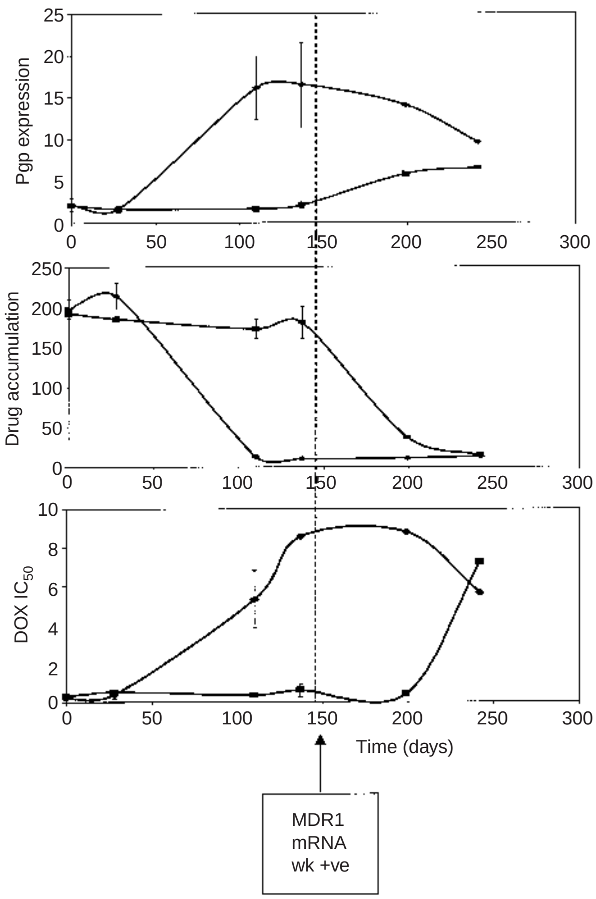

**Figure 4** Time course showing a significant delay in the emergence of the MDR1 phenotype in K562 cells after incubation in doxorubicin + dexrazoxane (rectangles) compared to doxorubicin alone (diamonds). Top panel: P-glycoprotein expression (ratio test/control); middle panel: drug accumulation (MFUs); bottom panel: doxorubicin $IC_{50}$

of both doxorubicin and dexrazoxane support this theory. Interestingly, to our knowledge, dexrazoxane has not been described as a modulator of MDR. Further investigating this possibility, we did not find any increase in doxorubicin sensitivity on short-term co-incubation with dexrazoxane in the chemosensitivity assay. Thus it appears that the positive effect of dexrazoxane is confined to the emergence of resistance rather than its circumvention. As dexrazoxane is known to cause cell cycle arrest at $G_2$/M (Sharpe et al, 1970; Scambia et al, 1995), it is possible that by slowing down the growth rate of these sublines, dexrazoxane is delaying mutation to the resistant phenotype.

PKC inhibitors such as staurosporine have been shown to block MDR1 induction by cytotoxic drugs (Chaudhary and Roninson, 1993). PKC-mediated transcriptional stimulation is thought to be exerted through the interaction of the Jun and Fos proteins with the AP-1 promoter element. A form of the AP-1 element is found in the major promotor of the human *MDR1* gene. Perhaps dexrazoxane also causes inhibition of PKC therefore blocking transcription of the *MDR1* gene. Recently Lucci et al (1999) reported that MDR modulators block the conversion of ceramide to glucosylceramide in MDR cells. Because ceramide is a critical component of the apoptosis-signalling cascade, this enhances cytotoxicity. It is possible that dexrazoxane can also affect cellular ceramide levels in cells exposed to MDR-related agents. These theories require further investigation.

K562 cells grown in doxorubicin + dexrazoxane may exhibit resistance caused by a mechanism other than overexpression of the *MDR1* gene hence the lack of functional P-glycoprotein. A similar study by Futscher et al (1996) using the human multiple myeloma cell line, RPMI 8226 (8226/S) demonstrated that cells selected by growth in doxorubicin alone exhibited the classical MDR phenotype mediated by the *MDR1* gene. However, drug resistance seen in doxorubicin + verapamil selected cells was mediated through decreases in topoisomerase II protein levels and catalytic activity and not by P-glycoprotein overexpression. The presence of another mechanism of resistance after growth in doxorubicin + dexrazoxane seems unlikely as we were unable to demonstrate any significant increase in doxorubicin $IC_{50}$ until after approximately 7 months continuous exposure to these agents in combination.

The question arises whether continuous exposure to low doses of these agents is clinically relevant, as conventional treatment with this combination commonly involves pulsed therapy every 3–4 weeks. Perhaps a more realistic experiment would be to mimic this pulsed therapy. Chaudhary and Roninson (1993), however, reported that MDR1 expression and associated resistance can continue for several weeks after the removal of the drug, suggesting that continuous exposure at low dose may be a reasonable model to test. Indeed, the delay in emergence of resistance seen in our model could translate into a considerable length of time in vivo with the pulsed therapy given in the clinic.

The original clinical observation of increased survival after therapy containing doxorubicin and dexrazoxane was made in breast cancer patients suggesting it would be very worthwhile repeating this in vitro study in the MCF7 human breast cancer cell line in the future. One of the reasons we used a leukaemia cell line for our study was because, in our experience, it is relatively easy to induce doxorubicin resistance in the K562 line to create an MDR-positive subline and we have previously used $K562DOX_{100}$ to control drug resistance experiments (Elgie et al, 1999). The other reason was that dexrazoxane has a proven effect in AML blasts, being cytotoxic after prolonged exposure (Pearlman et al, 1997). This group also showed that dexrazoxane does not abrogate the doxorubicin sensitivity of AML blasts in vitro. Indeed, dexrazoxane can potentiate the activity of other drugs, for example, topotecan in K562 cells (Synold et al, 1997) and also cisplatin in human ovarian cancer (Scambia et al, 1995). Lemez and Maresova (1998) found that 5 relapsed or refractory AML patients subsequently treated with daunorubicin + dexrazoxane all went into remission. Synold et al (1998) found that steady-state dexrazoxane plasma concentrations of 4 $\mu$M can be achieved safely and that prolonged exposure to dexrazoxane at this level is cytotoxic to human leukaemia cells in vitro. We did not find dexrazoxane to be cytotoxic at the nanomolar concentrations used in our study and there were no differences detected in the K562 sublines grown in dexrazoxane when compared to the parental line.

This is the first report of the ability of dexrazoxane to impair the emergence of drug resistance and these results may help to explain the observed increased median survival time found in responding breast cancer treated with FAC plus dexrazoxane. Anthracyclines are one of the most widely used groups of agents in chemotherapy and show significant efficacy in both haematological and solid tumours. Our observations suggest that the routine use of dexrazoxane in combination with anthracyclines may delay the onset of classical multidrug resistance, the major mechanism of resistance associated with these drugs. This would therefore enable patients who initially responded to their therapy to continue to respond

*© 2001 Cancer Research Campaign*

without the development of the most common obstacle to treatment failure. The clinical implications therefore, are immense and suggest that anthracycline-sensitive tumours should be treated with dexrazoxane ab initio if maximum benefit of the combination is to be obtained.

### ACKNOWLEDGEMENTS

This study was supported by the EB Hutchinson Trust, Tracy Sollis Leukaemia Trust, Edmund Fane Research Trust and the Round Table, Tunbridge Wells.

### REFERENCES

Advani R, Saba HI, Tallman MS, Rowe JM, Wiernik PH, Ramek J, Dugan K, Lum B, Villena J, Davis E, Paietta E, Litchman M, Sikic BI and Greenberg PI (1999) Treatment of refractory and relapsed acute myelogenous leukemia with combination chemotherapy plus the multidrug resistance modulator PSC 833 (Valspodar). *Blood* **93**: 787–795

Bordow SB, Haber M, Madafiglio J, Cheung B, Marshall GM and Norris MD (1994) Expression of the multidrug resistance-associated protein (*MRP*) gene correlates with amplification and overexpression of the N-*myc* oncogene in childhood neuroblastoma. *Cancer Res* **54**: 5036–5040

Chaudhary PM and Roninson IB (1993) Induction of multidug resistance in human cells by transient exposure to different chemotherapeutic drugs. *J Natl Cancer Inst* **85**: 632–639

Chomczynski P and Sacchi N (1987) Single-step method of RNA isolation by acid guanidinium thiocyanate-phenol-chloroform extraction. *Anal Biochem* **162**: 156–159

Elgie AW, Sargent JM, Williamson CJ, Lewandowicz GM and Taylor CG (1999) Comparison of P-glycoprotein expression and function with *in vitro* sensitivity to anthracyclines in AML. *Adv Exp Med Biol* **457**: 29–33

Futscher BW, Foley NE, Gleason-Guzman MC, Meltzer PS, Sullivan DM and Dalton WS (1996) Verapamil suppresses the emergence of P-glycoprotein-mediated multi-drug resistance. *Int J Cancer* **66**: 520–525

Hasinoff BB, Hellmann K, Herman EH and Ferrans VJ (1998) Chemical biological and clinical aspects of dexrazoxane and other bisdioxopiperazines. *Curr Med Chem* **5**: 1–28

Kato S, Ideguchi H, Muta K, Nishimura J and Natawa H (1990) Mechanisms involved in the development of adriamycin resistance in human leukemic cells. *Leuk Res* **14**: 567–573

Kaye SB (1999) New drug development: its role in reversing drug resistance. *Br J Cancer* **80**: 116–121

Lemez P and Maresova J (1998) Efficacy of dexrazoxane as a cardioprotective agent in patients receiving mitoxantrone-and daunorubicin-based chemotherapy. *Semin Oncol* **25**: 61–65

Lucci A, Han TY, Liu YY, Giuliano AE and Cabot MC (1999) Multidrug resistance modulators and doxorubicin synergize to elevate ceramide levels and elicit apoptosis in drug-resistant cancer cells. *Cancer* **86**: 300–311

Pearlman ML, Pagliaro LC, Liu B and Freireich EJ (1997) Dexrazoxane is cytotoxic to AML blasts and does not abrogate sensitivity to doxorubicin *in vitro*. *Proc Amer Assoc Cancer Res* **38**: 608, Abstr 4081

Sargent JM and Taylor CG (1989) Appraisal of the MTT assay as a rapid test of chemosensitivity in acute myeloid leukaemia. *Br J Cancer* **60**: 206–210

Scambia G, Della Bitta R, Benedetti Panici P, De Vincenzo R, Contu G, Ercoli A, Bonanno G, Pierelli L and Mancuso S (1995) Bisdioxopiperazine, (+)-1,2-Bis (3,5-dioxopiperazinyl-1-yl)propane (ICRF 187), enhances the antiproliferative effect of cisplatin on human ovarian cancer cells. *Gynecol Oncol* **57**: 16–22

Sharpe HBA, Field EO and Hellmann K (1970) Mode of action of the cytostatic agent 'ICRF 159'. *Nature* **226**: 524–526

Sikic BI, Fisher GA, Lum BL, Halsey J, Beketic-Oreskovic L and Chen G (1997) Modulation and prevention of multidrug resistance by inhibitors of P-glycoprotein. *Cancer Chemother Pharmacol* **40**: S13–S19

Swain SM, Whaley FS, Gerber MC, Ewer MS, Bianchine JR and Gams RA (1997) Delayed administration of dexrazoxane provides cardioprotection for patients with advanced breast cancer treated with doxorubicin-containing therapy. *J Clin Oncol* **15**: 1333–1340

Synold T, Spencer M and Doroshow J (1997) Dexrazoxane (DX) potentiates the cytotoxic activity of topotecan (T) in a sequence and schedule dependent manner. *Proc Amer Assoc Cancer Res* **38**: 322, abstr 2161

Synold TW, Tetef ML and Doroshow JH (1998) Antineoplastic activity of continuous exposure to dexrazoxane: potential new role as a novel topoisomerase II inhibitor. *Semin Oncol* **25**: 93–99

Trock BJ, Leonessa F and Clarke R (1997) Multidrug resistance in breast cancer: a meta-analysis of MDR1/gp170 expression and its possible functional significance. *J Natl Cancer Inst* **89**: 917–931

Weiss RB (1999) The randomized trials of dose-intensive therapy for breast cancer: What do they mean for patient care and where do we go from here? *Oncologist* **4**: 450–458

Yanagisawa T, Newman A, Coley H, Renshaw J, Pinkerton CR and Pritchard-Jones K (1999) BRICODAR (VX-710; Incel™): an effective chemosensitizer in neuroblastoma. *Br J Cancer* **80**: 1190–1196

© 2001 Cancer Research Campaign
